# Ultrasound powered piezoelectric neurostimulation devices: a commentary

**DOI:** 10.1186/s42234-020-00052-6

**Published:** 2020-08-12

**Authors:** Tao Sun, Jason Wright, Timir Datta-Chaudhuri

**Affiliations:** 1 Institute of Bioelectronic Medicine, The Feinstein Institutes for Medical Research, Manhasset, NY USA; 2 Zucker School of Medicine at Hofstra/Northwell, Hempstead, NY USA

**Keywords:** Piezoelectric neural stimulator, Spinal cord injury, Ultrasound, Wireless power, Implantable electronics, Neuromodulation

## Abstract

Conventional neurostimulation systems for preclinical research can be bulky and invasive due to the need for batteries or wired interfaces. Emerging as a new neural interface technique, ultrasound-powered piezoelectric neural stimulators work by converting ultrasound energy to electrical charge for neural stimulation. In addition to the benefits of wireless powering and miniaturization leading to less traumatic surgery, piezoelectric neural stimulators can also exhibit prolonged operational lifetimes for a long-term stable neural interface, and show promise for clinical translation. As one of first steps to demonstrate the value of ultrasound-powered piezoelectric neural interface, Li et al. developed a piezoelectric stimulator to activate spinal cord neural circuits for locomotion restoration in a rat model with spinal cord injury (SCI) and compared its efficacy with conventional electrical stimulation (ES). From the point of view of materials science, neural engineering and microelectronics, we provide our commentary on the article, highlighting its importance and discussing the issues that remain to be addressed in future studies in the emerging field of ultrasound powered piezoelectric neurostimulation devices.

## Background

Most active electronic medical implants today utilize onboard batteries as their power source. The necessity for periodic battery replacement not only constrains the lifetime of the medical implants, but also requires further surgeries that result in additional trauma for patients. For example, a non-rechargeable battery used for a deep brain stimulator was reported to have a lifetime of 4 to 5 years, according to an investigation with 192 patients (Helmers et al. 2018). Consequently, many wireless power transfer techniques have emerged as alternative approaches for providing energy for device operation, including inductive coupling, ultrasound, radio frequency and heat (Taalla et al. 2019). The use of ultrasound to power medical devices has benefits of volume reduction of implants (down to mm dimensions) (Charthad et al. 2015), operation across longer depths of tissue (~ 10 cm) (Charthad et al. 2018), and moderate power transfer efficiency (~ 40%, depending on working distance) (Ozeri et al. 2010). Wireless powering technologies for clinical applications are mature and utilized in many marketed devices, but the issue of providing long lasting power for preclinical devices is of particular importance because batteries do not scale as readily as electronics, and studies utilizing small animal models have significant constraints on device size and mass. In addition to the work by Li et al. *(*Li et al. 2020*)*, here we also examine another recently developed neural stimulation system powered by ultrasound, a smaller millimeter-scale device used to interface the sciatic nerve with bidirectional communication capability (Piech et al. 2020). These two examples allow us to compare some of the design tradeoffs of different implementations of this emerging technology.

Traumatic spinal cord injury (tSCI), mostly caused by accidents, severs the signal flow between brain and body systems, resulting in as-yet irreversible loss of functions, such as paralysis. Globally, it is estimated that more than 27 million patients are living with long-term disability due to SCI, while in North America alone there are 12,500 new cases of SCI each year (Hachem et al. 2017; Bradbury and Burnside 2019). Etiologically, tSCI is the most common form and accounts for more than 90% of SCI cases (James and Theadom 2019). In spite of the recent progress in neuroscience and biomedical engineering, there has been no effective therapy to regenerate adult central nervous system axons and repair the spinal cord pathways after severe SCI (de Cassia Sampaio et al., 2016). Epidural spinal cord stimulation shows promise to promote and restore voluntary movement, after chronic neurologically complete SCI (Darrow et al. 2019).

**Fig. 1:**
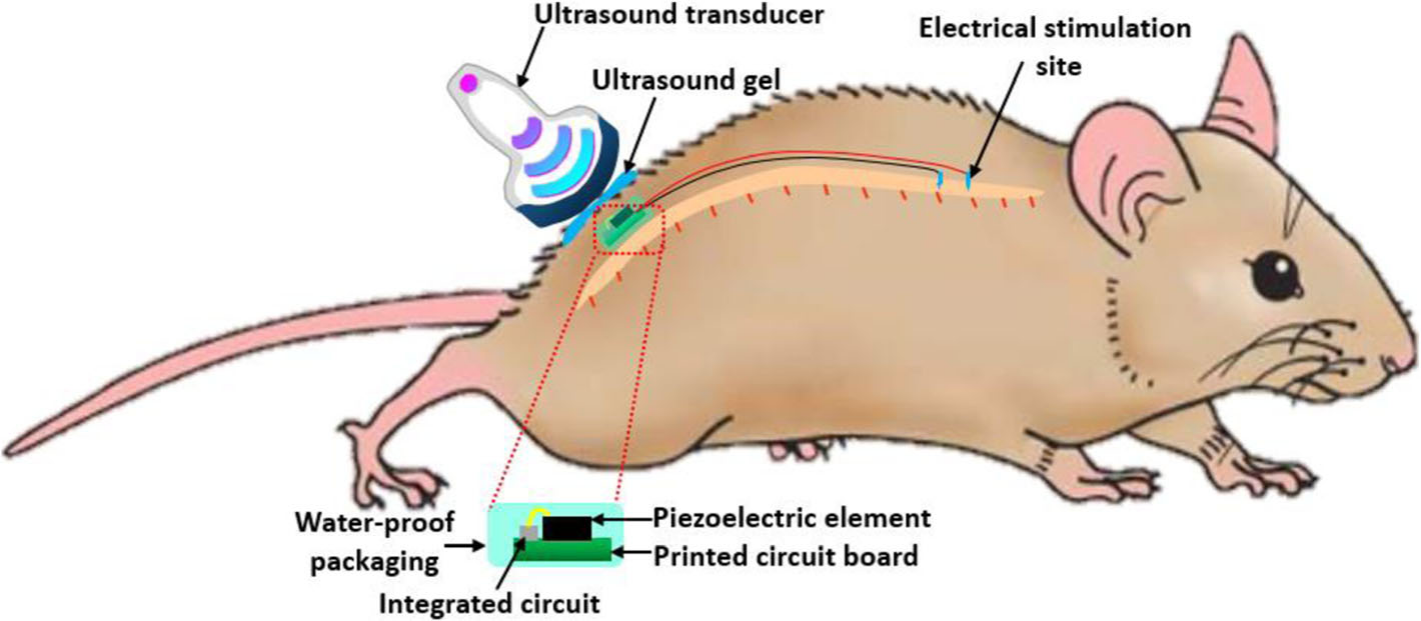
Working principle of the ultrasound-driven piezoelectric stimulator interfacing the spinal cord for the restoration of involuntary locomotion. The ultrasound energy provided by the probe is converted to electrical energy for neural stimulation by the implanted device

In the article published in Bioelectronic Medicine, Li et al. proposed an ultrasound-driven barium titanate (BaTiO_3_) piezoelectric stimulator for restoration of involuntary locomotion in rats with SCI by means of epidural spinal cord stimulation. Figure 1 shows the working principle of the ultrasound-driven piezoelectric stimulator interfacing the spinal cord for the restoration of involuntary locomotion. Similar to conventional electrical stimulation (ES), the piezoelectric current generated from the ultrasonic power transmission activates spinal cord neural circuits and enables paralyzed rats to move their hind legs. Despite the fact that many technical challenges (such as long-term reliability in usage, need for precise alignment between the transceiver and the implant, integrated circuit design for higher power conversion efficiency, etc.) are yet to be addressed for the development of robust ultrasonic-powered stimulation micro-systems, we optimistically consider the technique as a promising new avenue for neuromodulation in the field of bioelectronic medicine.

## Commentary

The concept of the piezoelectric stimulators has been extensively studied (Piech et al. 2020; Phillips et al. 2003; Marino et al. 2017; Alam et al. 2019), but their efficacy has not been established in comparison with conventional ES in terms of the restoration of involuntary locomotion. The work by Li et al. provides further insights to fill in the gap and demonstrate that piezoelectric stimulation (pES) without a battery can achieve comparable efficacy to ES. In this commentary, we discuss the article details with emphasis on the technical aspects of implementing such systems.

### Alignment and position

To effectively deliver acoustic energy for the piezoelectric stimulator, accurate alignment and positioning between the ultrasound transducer and piezoelectric stimulator are critical during nerve stimulation. Piezoelectric devices convert mechanical displacement into electrical charge, and because they are comprised of a regularly repeating crystalline structure they are sensitive to the relative angle of applied mechanical energy. Ultrasound energy is also attenuated with distance with a factor of attenuation which depends on the materials that it passes through. In general this requires a greater amount of energy to be generated at the source compared to what is received at the implanted device. However, there are techniques such as focusing which can be used concentrate the energy at the implant location while applying lower ultrasound energy density over a larger skin contact area.

### Piezoelectric stimulator dimensions

One of the potential benefits of utilizing ultrasound to power a piezoelectric implant is that it obviates the necessity for a battery within the implantable device. Given that batteries occupy a large percentage of the volume of implantable devices, this enables the design of devices that are significantly smaller in size than those requiring batteries. In the article by Li et al., the diameter of the piezoelectric stimulator employed was 10 mm with a height of 4 mm (Alam et al. 2019) yielding a volume of 314 mm^3^. Compared to some commercial button cells (5.8 mm in diameter and 1.6 mm), the size of the piezoelectric stimulator is still relatively large and could benefit from further shrinking so that there will be more options to implant the device into anatomical pockets of small animals. However, this should not be considered an issue for the applicability of piezoelectric stimulators since mm scale implants have already been demonstrated (a recent example had a volume of 1.7 mm^3^ (Piech et al. 2020)).

### Ultrasound biosafety considerations

All radiation exposure presents intrinsic safety and health risk considerations due to the potential for tissue damage. However, ultrasonic power transfer is widely used in diagnostic imaging, and its risks are managed by limiting the transmitted acoustic power density to a safe amount. The maximum FDA limit on ultrasound exposure is 720 mW/cm^2^ spatial-peak temporal-average intensity (I_SPTA_) and 190 W/cm^2^ spatial-peak pulse-average intensity (I_SPPA_) (Marketing Clearance of Diagnostic Ultrasound Systems and Transducers 2019).

The transducer used by Li et al. outputs a maximum of 22.5 mW/cm^2^ (I_SPTA_) at 40 Hz and 3.9 W/cm^2^ (I_SPPA_), significantly below FDA limits. Although safe exposure limits for rodents are not generally well established, histological analysis from previous work suggests no negative effects at this level of exposure (Kim et al. 2014). The size of the piezoelectric component plays a role in the amount of power that can be effectively transferred. The power transfer efficiency of the piezoelectric stimulator in this work was approximately 0.22%, from the input power to the driving transducer to received electrical power at the stimulator. The significantly smaller design employed in (Piech et al. 2020) had similar efficiencies of .33% in ex vivo tissue and .7% in a gel phantom. However, the smaller design required substantially higher amounts of ultrasound power to be generated. This work showed that intensities as low as 0.1 mW/cm^2^ could induce motor evoked potentials and that an intensity of 22.5 mW/cm^2^ could be used to induce locomotion in hind limbs. The smaller design in (Piech et al. 2020) required a minimum intensity of 56 mW/cm^2^ at optimal orientation, alignment, distance; and required 451 mW/cm^2^ at a distance of 55 mm within ex vivo tissue. It should be noted that the FDA limit for diagnostic ultrasound exposure of 720 mW/cm^2^ as stated above is only for peripheral vessels, and the limits range down to 17 mW/cm^2^ for ophthalmic tissue. This indicates that although it is possible to significantly shrink such devices, there exists a tradeoff between device size and the amount of power that can safely be transferred to the device using ultrasound. Translation to clinical applications would require additional considerations, as limits differ for other organs in the body and by operating frequency. Additionally, threshold intensity will depend on acoustic impedance of surrounding tissue and acoustic mismatch of tissue interfaces, which may differ in clinical applications.

### Encapsulation strategies

In their work, Li et al.*,* encapsulated the piezoelectric stimulator using a biocompatible silicone coating. The lifetime and long-term reliability of the electrically packaged stimulator remains unknown in the in vivo environment and requires further longitudinal studies. To reduce the fibrous tissue encapsulation surrounding the piezoelectric stimulator and the resulting increase in acoustic impedance, packaging techniques utilizing clinically proven long-lasting biocompatible materials need to be developed and validated. Other approaches have used a conformal coating of Parylene to encapsulate the device (Piech et al. 2020), which according to the authors was expected to last for durations of months to years. Although Parylene is known to be biocompatible and appealing because it can be deposited in very thin layers, its longevity in biological environments remains poor ever since it was first evaluated in the 1970s (Barrese et al. 2013; Schmidt et al. 1988; Loeb et al. 1977). The degradation of Parylene encapsulated structures is primarily due to poor chemical bonding to underlying substrates (Stieglitz et al. 2002) and moisture absorption which lead to delamination and cracking over time. These issues are further exacerbated when the Parylene is selectively removed to expose active sites such as electrodes which must contact the tissue, as this results in an exposed interfacial surface underneath the Parylene which presents an opportunity for moisture ingress. Silicone encapsulation is appealing because it is compliant and can be used to minimize the foreign body response to the implant, however it suffers from similar issues as Parylene including moisture absorption and poor adhesion to underlying materials such as insulated wires leading to electrodes. Another approach to polymer-based packaging has been to incorporate layers of different polymers such as Parylene and epoxy (Wright et al. 2019) to try to benefit from the different properties of the different materials, but the addition of additional encapsulation layers and the resulting added thickness can result in higher acoustical impedance and lower power transfer efficiency for ultrasound powered devices.

### Comparison with conventional electrical stimulation

The efficacy of pES was rarely reported in systematic comparison with ES. In this study, the efficacy of the piezoelectric stimulator was validated by comparing motor evoked potentials in the hindlimb muscles triggered by both epidural electrical stimulation and piezoelectric stimulation. As evidenced by similar recruitment of tibialis anterior muscles for ES and pES, the efficacy of pES is comparable to conventional ES. Likewise, both ES and pES rendered rats with SCI to regain the hindlimb locomotion on a moving treadmill belt, indicating that the performance of ES and pES is likely to be consistent. However, this exploratory study utilized seven rats; data generated from one rat was displayed in two figures (Figures 2d and 4 in the article), but no formal hypothesis test was employed with statistical analysis. Additional data and a more comprehensive statistical analysis are needed to conduct an efficacy comparison between ES and pES.

While this article focuses on the effects of pES, ultrasound exposure can also stimulate neuronal circuits, and its effects have been established in studies spanning multiple species at varying frequencies and intensity levels (Tufail et al. 2011). In (Cotero et al. 2019) it was shown that ultrasound pressures well within the limits for diagnostic imaging were successfully able to stimulate substructures within organs resulting in changes in production of inflammatory markers in a manner similar to stimulating the vagus nerve (Pavlov and Tracey 2012). Further studies are required to understand the possibility of transferring ultrasound energy to implants in a manner which does not disturb normal operation of the biological systems in the body. Importantly, pES should be compared to sham stimulation (in which the same transducer setup is used in conjunction with a “dummy” implant) to isolate the effects of pES from the effects of ultrasound exposure.

### Wireless stimulation

Neural stimulation systems are either wireless or wired. Both type of systems require an implanted electrode to interface to the nervous system. Wired systems require physical wired connections from the implanted device to an external benchtop system providing either stimulation pulses or power for electronics within the device. Wireless systems incorporate stimulation generation within the implanted device and can be powered using either internal energy storage such as a battery or capacitor, or by wirelessly receiving power from an external power source. Wired systems require handling of the animal to attach the wires, and once attached, they limit the movement of the animal, leading to trauma and restriction of the animal’s normal behavior. Additionally, the percutaneous connectors used for wired systems present a lasting opportunity for infection and irritation to the animal. Truly wireless systems do not have these issues since they can be controlled and powered wirelessly.

Ultrasound-based powering of implantable devices is generally considered to be part of a wireless system, but may not provide all of the benefits of wireless implants outside of the lack of a percutaneous connection system. Ultrasound power is significantly attenuated in air, and proper power transfer for the types of systems described here requires intimate contact between the ultrasound transducer and the skin. This additionally requires restraining and possibly anesthetizing the animal, which can be a source of trauma and change the physiological state of the animal. This appears to be a fundamental limit of ultrasound powered systems; to our knowledge there is no ultrasound powered implant which does not require intimate contact between the power source and the animal.

## Conclusion

This research shows promising results to restore involuntary locomotion for rats with SCI, using a novel piezoelectric stimulator. The authors compared the motor evoked potentials in the hind limb muscles response to epidural electrical stimulation and piezoelectric stimulation, and restored the hind limb locomotion for rats with SCI by these two approaches. Eventually, this study demonstrated that there was no notable difference between epidural electrical stimulation and piezoelectric stimulation in terms of motor evoked potentials and involuntary locomotion restoration, laying a solid foundation to prove the value for pES.

## Data Availability

Not applicable.

## Abbreviations

SCI: spinal cord injury
ES: electrical stimulation
tSCI: traumatic spinal cord injury
pES: piezoelectric stimulation
FDA: US Food & Drug Administration
